# The nutritional status of people with alkaptonuria: An exploratory analysis suggests a protein/energy dilemma

**DOI:** 10.1002/jmd2.12084

**Published:** 2020-03-17

**Authors:** Shirley Judd, Milad Khedr, Anna M. Milan, Andrew S. Davison, Andrew T. Hughes, Alexander Needham, Eftychia E. Psarelli, Alan Shenkin, Lakshiminaryan R. Ranganath

**Affiliations:** ^1^ Department of Nutrition and Dietetics Royal Liverpool University Hospital Liverpool UK; ^2^ Department of Clinical Biochemistry and Metabolic Medicine Liverpool Clinical Laboratories, Royal Liverpool University Hospital Liverpool UK; ^3^ Liverpool Cancer Trials Unit University of Liverpool, Block C, Waterhouse Building Liverpool UK

**Keywords:** alkaptonuria, global nutritional assessment, protein energy deficit

## Abstract

**Background:**

Alkaptonuria (AKU) is a disorder of tyrosine/protein metabolism leading to accumulation of homogentisic acid. Clinical management historically recommended reducing dietary protein intake, especially in childhood, which has since been discredited in the literature. For the first time, analysis of baseline cross‐sectional nutritional surveillance data from a large cohort of AKU patients is presented, which has clinical implications in all aspects of treatment planning.

**Method:**

Seventy‐four patients (mean 55 years) admitted to the National Alkaptonuria Centre (NAC), underwent a global nutritional assessment, which included objective anthropometry, bioimpedance measures, habitual nutritional intake using a 7‐day food diary and key nutritional biomarkers, including 24 hours urinary nitrogen, serum albumin, total protein and total 25‐hydroxy vitamin D. All data was compared with cohort norms or recommended nutrient intakes for health (RNI). The potential beneficial impact of protein and anti‐inflammatory nutrients such as vitamin C, selenium, and zinc were statistically interrogated against the AKU severity score index (AKUSSI)—a validated measure of disease progression stratified by age.

**Results:**

Fifty percent of AKU patients reported some level of protein restriction at some point in their lives. In comparison with national data sets, AKU patients present with significantly lower than predicted mid‐upper arm circumference, grip strength, BMI, total energy and protein intake, and higher than predicted percentage body fat. They therefore meet the ESPEN criteria as “clinically undernourished.” Severity fluctuates over the life course. No statistical association is identified between protein intake, expressed as %RNI or g/kg, or anti‐inflammatory nutrients, including vitamin C as a high dose supplement on the severity of the disease, when correlated against the validated AKUSSI score.

**Conclusion:**

AKU patients are at risk of protein depletion associated with a “perfect storm” of risk factors: historical, poorly evidenced recommendations to reduce total protein intake; limited mobility as the condition progresses, compromising muscle integrity; frequent hospital admissions for major surgery associated with multiple joint replacements, creating pinch points of high metabolic demand and the potential impact of the disease itself. As this is the first time this risk has been identified, the authors consider the dietetic implications of nitisinone treatment, which requires dietary protein control to manage the acquired tyrosinaemia. The lack of statistically significant evidence to support dietary manipulation of any kind to impede disease progression in AKU is demonstrated.

**SYNOPSIS**

Adults with AKU are likely to be clinically under‐nourished, exacerbated by frequent surgical interventions over their life course. Management of nitisinone‐induced tyrosinaemia, potentially exacerbates this. Specialist dietetic support is needed to safeguard patients' nutritional status.

## 1 INTRODUCTION

Alkaptonuria (AKU, OMIM#203500), results from mutations in the homogentisate 1,2 dioxygenase gene (HGD, EC 1.13.11.5),^1^ increasing circulating homogentisic acid (HGA) and urinary HGA, an intermediary metabolite in the catabolism of the amino acids phenylalanine and tyrosine. HGA oxidizes to benzoquinoneacetic acid (BQA) that deposits ochronotic pigment in soft tissues, cartilage and connective tissue, most typically causing severe arthritis of weight bearing joints and spine.^2^

With no proven disease modifying therapy for AKU, treatment approaches were historically supportive (physiotherapy and analgesia) and palliative (spinal surgery and joint replacement). There is no definitive dietary treatment recommended for AKU; however, limiting protein intake in childhood^3^ and high dose ascorbic acid^4^, ^5^ are two cited dietary strategies.

This report presents an overview of the nutritional status of patients with AKU at the initial baseline visit to the UK National AKU Centre (NAC), which was established in 2012. The overall nutritional status of this cohort is presented and asks if there is an association between habitual food intake and the severity of the condition, using the validated numerical AKUSSI score.^6^ The dietetic implications for supporting the NAC's treatment protocol of providing HGA lowering therapy using off license 2 mg nitisinone/day are then considered.

There is little published evidence to guide the use of a lower tyrosine intake to modify AKU disease progression or minimize the consequences of postnitisinone tyrosinaemia. It is thus crucial to more fully understand the role diet plays in AKU.

## 2 METHODS

### 2.1 Study population

Forty‐seven males (64%) and 27 females (36%) with a median age of 55 (range 17‐72 years) and a confirmed diagnosis of AKU were included in the study. These 74 patients attended the NAC from 2012 to 2018.

Patients underwent a global nutritional assessment, which included objective anthropometry (Table 1), habitual nutritional intake using a 7‐day food diary (Table 2) and nutritional biomarkers, including 24‐hours urinary nitrogen and serum albumin, total protein, 25‐hydroxy vitamin D and hemoglobin (Table 3). The findings were compared with key national reference sources, the National Diet and Nutrition Survey^7^ and current UK recommendations for health—The Eat well Guide (http://www.gov.uk;^8^). Statistical comparisons were made with the age and sex specific UK Reference Nutrient Intakes (RNI's) for each nutrient^9^, ^10^ and body composition reference data used in routine clinical practice.^11^

**Table 1:** Body composition

Variable	Number	Median	IQR	Mean	SD	Nearest centile to the mean
1(a)						
MUAC (cm)	74	29.9	27.0‐32.5	30.0	3.8	
Male	47	30.0	28‐32.5	30.2	3.2	15
Female	27	29.0	26.0‐32.5	29.6	4.7	Mid‐point of 25th and 50th
Males (age)						
20‐29	7	32.5	25.5‐34.0	30.7	4.0	15
30‐39	5	31.5	28.6‐32.0	30.7	2.1	15
40‐49	12	31.6	29.4‐33.0	31.1	2.7	15
50‐59	12	29.2	28.0‐31.0	29.9	3.6	15
60‐69	7	29.0	26.6‐32.3	28.9	3.1	10
70‐79	2	29.1	25.6‐32.5	29.1	4.9	15
Females (age)						
20‐29	0	–	–	–	–	–
30‐39	5	29.0	27.0‐33.4	31.7	7.2	50
40‐49	3	26.0	24.6‐32.4	27.7	4.2	15
50‐59	5	30.0	29.0‐30.5	31.4	4.6	50
60‐69	10	31.1	28.0‐32.5	30.1	2.9	25
70‐79	1	25.0	25.0‐25.0	25.0		15

Variable	Number	Median	IQR	Mean	SD
1(b)					
Muscle mass (kg)	49	51.4	42.4‐55.9	49.2	9.0
Male	34	39.3	50.2‐58.3	53.8	6.5
Female	15	53.6	36.2‐42.4	39.0	4.4
Males (age)					
19‐64	29	54.5	51.9‐58.7	54.8	6.3
≥65	4	46.9	43.4‐50.5	47.0	4.4
Females (age)					
19‐64	10	40.2	36.2‐42.8	40.1	4.2
≥65	5	36.6	35.2‐39.3	36.8	4.4

Variable	Number	Median	IQR	Mean	SD	Normal/85%
1(c)						
Grip strength (kg)	49	29.8	22.0‐35.5	29.7	13.2	
Male	34	33.8	26.3‐39.1	33.1	13.3	40/34
Female	15	21.0	17.2‐24.9	22.0	9.2	27.5/23
Males (age)						
18‐69	32	33.8	26.2‐39.2	33.2	13.7	40/34
70‐79	2	31.7	29.1‐34.3	31.7	3.7	32.5/27.5
Females (age)						
18‐69	14	20.2	17.2‐24.6	21.8	9.5	27.5/23
70‐79	–	n/a	n/a	n/a	n/a	25.0/21

Variable	Number	Median	IQR	Mean	SD	Nearest centile to the mean
1(d)						
% Body fat (50 kHz BIA)	49	25.2	22.0‐30.9	26.2	9.3	
Male	34	24.6	20.3‐28.5	24.8	8.8	75
Female	15	28.2	24.1‐35.0	29.5	9.8	50
Males (age)						
18‐24	2	6.6	5.7‐6.6	6.6	1.2	5
25‐34	3	23.0	21.0‐51.2	31.7	16.9	>95th
35‐44	8	25.3	20.2‐33.7	27.0	8.7	90
45‐54	11	26.0	23.1‐28.5	26.2	4.6	90
55‐64	6	24.8	20.3‐26.6	23.8	4.9	50
65‐74	4	20.6	15.1‐28.9	22.0	8.8	25
Females (age)						
18‐24	1	25.9	25.9‐25.9	25.9	–	50
25‐34	2	30.9	23.0‐38.8	30.9	11.2	75
35‐44	3	28.2	24.1‐30.9	27.7	3.4	50
45‐54	2	16.7	8.6‐24.7	16.7	11.4	5
55‐64	2	34.5	26.3‐42.6	34.5	11.5	50
65‐74	4	33.3	31.3‐42.5	36.9	8.9	25

Variable	Number	Median	IQR	Mean	SD	England mean 2014
1(e)						
BMI (kg/m^2^)	72	26.8	23.0‐29.0	26.7	5.2	24.1
Male	45	26.9	24.8‐29.0	26.9	4.3	27.2
Female	27	25.7	21.3‐29.0	26.3	6.6	27.2
Males (age)						
18‐24	4	22.4	19.2‐26.5	22.8	4.5	23.8
25‐34	6	25.6	24.8‐28.9	25.5	3.7	26.4
35‐44	10	26.8	25.5‐31.1	28.1	4.8	27.7
45‐54	12	27.8	25.3‐30.5	28.0	4.2	28.4
55‐64	8	27.0	25.9‐27.9	27.2	3.0	28.9
65‐74	5	29.2	20.8‐30.1	26.5	5.5	27.9
Females (age)						
18‐24	2	20.5	19.9‐21.0	20.5	0.8	24.4
25‐34	3	29.9	21.0‐47.7	32.9	13.6	26.2
35‐44	4	23.3	22.0‐26.5	24.2	3.3	27.2
45‐54	2	20.5	17.4‐23.6	20.5	4.4	28.1
55‐64	9	28.8	25.7‐30.0	27.9	5.3	28.6
65‐74	6	27.2	23.6‐28.1	27.3	4.2	28.4

Variable	Number	Median	IQR	Mean	SD	England BMI mean 2014
1(f)						
BMIPTH	74	25.2	22.5‐28.3	25.6	4.1	24.1
Male	47	25.5	23.5‐28.1	25.5	3.33	27.2
Female	27	24.9	21.7‐29.2	25.8	5.21	27.2
Males (age)						
18‐24	4	22.9	20.1‐26.3	23.2	3.6	23.8
25‐34	6	26.2	23.5‐28.7	26.2	4.5	26.4
35‐44	10	26.8	24.1‐28.3	26.5	2.8	27.4
45‐54	13	26.4	23.5‐27.5	26.0	3.7	28.4
55‐64	8	24.8	23.5‐25.7	24.4	2.4	28.9
65‐74	6	24.4	22.4‐28.5	25.0	3.0	27.9
Females (age)						
18‐24	2	20.7	19.2‐22.1	20.7	2.1	24.4
25‐34	3	29.4	22.5‐40.6	30.8	9.1	26.2
35‐44	4	22.9	20.6‐26.7	23.6	3.9	27.2
45‐54	2	24.0	21.7‐26.3	24.0	3.3	28.1
55‐64	9	25.2	24.1‐29.2	25.9	5.5	28.6
65‐74	7	27.7	24.4‐29.4	26.8	3.4	28.4

**Table 2:** Nutritional intake

Variable	Number	Median	IQR	Mean	SD	NSDS mean
Energy (kcal)	72	1679.0	1397.5‐2013.0	1730.9	455.0	1849
Male	46	1883.0	1459.0‐2227.0	1856.7	454.4	1971
Female	26	1452.0	1244.0‐1684.0	1508.4	368.3	1542
Males (age)						
19‐64	39	1907.0	1519.0‐2313.0	1910.4	465.4	2107
≥65	6	1452.5	1360.0‐1674.0	1531.0	242.2	1835
Females (age)						
19‐64	19	1466.0	1143.0‐1835.0	1544.7	422.9	1595
≥65	7	1438.0	1296.0‐1519.0	1409.6	114.0	1488
Fe %RNI	71	105.0	77.0‐130.0	106.6	38.3	Mean %RNI
Male	45	113.0	88.0‐139.0	116.6	33.4	130
Female	26	87.0	58.0‐110.0	89.4	40.7	91
Folate %RNI	71	100.0	75.0‐134.0	106.0	39.9	Mean %RNI
Male	45	107.0	77.0‐138.0	111.8	41.5	135
Female	26	87.0	75.0‐110.0	95.9	35.4	108
Ca %RNI	71	105.0	85.0‐151.0	115.7	42.6	Mean %RNI
Male	45	126.0	88.0‐158.0	125.7	44.0	125
Female	26	94.5	76.0‐109.0	98.5	34.3	107
Fiber g(NSP) aim 18 g	72	12.5	9.0‐16.0	13.7	6.2	14
Male	46	13.0	10.0‐17.0	14.5	6.7	15
Female	26	11.5	8.0‐15.0	12.3	4.9	13
%Protein/RNI	72	123.5	104.0‐150.5	128.9	34.3	
Male	46	125.5	105.0‐157.0	131.2	35.2	
Female	26	120.0	96.0‐143.0	124.7	33.1	
Males (age)						
19‐64	39	125.0	104.0‐157.0	129.7	36.0	
≥65	6	124.5	122.0‐131.0	130.0	19.9	
Females (age)						
19‐64	19	120.0	95.0‐148.0	125.1	36.4	
≥65	7	122.0	110.0‐143.0	123.4	24.1	
Protein (g)/day	71	70.0	55.0‐85.0	67.5	20.7	74.4
Male	45	75.0	62.0‐89.0	75.7	22.0	
Female	26	60.5	51.0‐70.0	66.6	14.6	
Males (age)						
19‐64	38	76.5	58.0‐91.0	75.7	23.7	84.6
≥65	6	73.5	67.0‐76.0	71.0	10.0	74.4
Females(age)						
19‐64	20	58.5	51.0‐77.0	67.4	15.8	64.4
≥65	6	60.6	57.0‐61.0	58.7	9.9	64.3
Selenium (%RI)	74	56.0	38.0‐72.0	55.4	21.5	72
Male	45	56.0	36.0‐75.0	56.0	22.2	
Female	27	56.0	39.0‐60.0	53.7	19.0	
Males (age)						
19‐64	39	56.0	40.0‐75.0	55.9	20.6	72
≥65	5	31.0	28.0‐86.0	53.6	36.4	68
Females(age)						
19‐64	19	53.0	38.0‐60.0	54.0	21.7	72
≥65	8	57.5	48.5‐60.0	53	11.2	70
Zinc (%RI)	74	88.0	70.0‐108.0	90.8	27.9	103
Male	45	92.0	81.0‐108.0	94.6	25.2	
Female	27	72.0	62.0‐105.0	83.4	31.3	
Males (age)						
19‐64	39	95.0	80.0‐119.0	95.0	26.9	101
≥65	5	90.0	87.0‐101.0	92.2	11.5	93
Females(age)						
19‐64	19	70.0	60.0‐105.0	82.7	35.1	106
≥65	8	84.0	70.0‐106.5	85.3	21.7	108
Vitamin C (%RNI)	74	185.5	101.0‐268.0	190.1	104.7	
Male	45	200.0	103.0‐267.0	190.8	108.5	
Female	27	164.0	100.0‐278.0	185.1	97.4	
Males (age)						
19‐64	39	219.0	103.0–267.0	199.7	108.9	
≥65	5	108.0	104.0‐201.0	149.8	94.8	
Females (age)						
19‐64	19	210.0	100.0‐290.0	194.7	103.4	
≥65	8	162.0	105.5‐223	162.4	83.2	

**Table 3:** Comparing estimated protein intakes from the 7 days food diary and 24 hours urinary nitrogen (g/kg body weight)

Variable	Number	Median	IQR	Mean	SD
Food diary	72	1.0	0.8‐1.2	1.0	0.3
Male	46	1.0	0.8–1.2	1.0	0.3
Female	26	0.9	0.8‐1.1	1.0	0.3
Males (age)					
19‐64	39	1.0	0.8–1.2	1.0	0.3
≥65	6	1.0	0.9‐1.1	1.0	0.2
Females (age)					
19‐64	19	0.9	0.8‐1.3	1.0	0.4
≥65	7	1.0	0.8–1.1	1.0	0.2
Urinary *N*	56	0.9	0.7‐1.0	0.9	0.2
Male	36	0.8	0.7–1.0	0.8	0.2
Female	20	0.9	0.7–1.0	0.9	0.2
Males (age)					
19‐64	31	0.8	0.7–1.0	0.9	0.2
≥65	4	0.8	0.6‐1.0	0.8	0.3
Females (age)					
19‐64	14	0.9	0.7‐1.1	0.9	0.3
≥65	6	0.9	0.7–1.0	0.9	0.2

### 2.2 Body composition assessment

This was undertaken using standard techniques.^7^ Objective data included Mid Upper Arm Circumference (MUAC in cm) and nondominant arm grip strength (kg). A digital dynamometer (model MG4800, Marsden Weighing Scales, Ltd.) was used for all measurements, undertaken in a consistent sitting position, specifically chosen for its lightness, for this patient group, as part of a longitudinal approach. Data from those patients reporting joint pain in the hand associated with the AKU condition were excluded from the analysis, most commonly the thumb joint affecting ability to grip the device.

Weight, muscle mass, and % body fat/fat mass (kg) were assessed using bioimpedance measurements (Tanita scale model DC‐430S MA) from 2013 onwards. Height was taken, using a wall‐mounted stadiometer (Seca 222) accurate to the nearest 0.1 cm. Weight was taken in light clothing to the nearest 0.1 kg. Body mass index (BMI) was calculated as the ratio of weight in kg to the square of height in meters as defined by WHO^12^ and for comparative purposes the Powell‐Tuck and Hennessy equation,^13^ which estimates BMI using MUAC (referred to as a BMIPTH) was calculated, given height loss and spine curvature is common in this condition. MUAC and grip strength readings were compared to USA National Health & Nutritional Examination Survey (NHANES)^14^ by age and sex, due to the lack of UK centiles covering the entire adult population, as is standard professional practice. Bioimpedance markers were compared to national centiles.^15^, ^16^

The data has been collected and analyzed by the same dietitian from November 2012 (SJ) with the exception of three patients whose anthropometric data was undertaken by an experienced dietetically trained assistant.

### 2.3 Habitual food intake

This was assessed using a 7‐day diet diary, completed the week before. In a service standardized format, the diary includes completion notes, a series of questions about usual eating habits, portion sizes, cooking methods and in the case of manufactured goods, named brands (Supporting Information, Data S1). Data was coded using standardized techniques and entered into computerized analysis databases (Microdiet version 3, from 2012 to 2014), and nutritics (using Microdiet version 8 from 2015 onwards^17^). If weights were not given, portion sizes were interpreted using Foods Standards Agency norms.^18^

Results were compared to UK Dietary Reference Values (DRV's) adjusted for age and sex, interpreted by relative nutritional risk, as a percentage of target for each nutrient's recommended nutrient intake (RNI) and lower recommended nutrient intake (LRNI) to denote deficiency risk. Total protein intake was compared to COMA^9^ and SACN^19^, ^20^, ^21^ and presented as g/kg body weight. Overall, nutrient intakes were compared with the National Diet and Nutrient Survey^7^ to assess potential differences against national profiles, for the relevant years of study.

### 2.4 Measurements of nutritional parameters in blood and urine

This included fasting serum albumin (g/L), total protein (g/L), hemoglobin (g/L), and the total 25‐hydroxy vitamin D (Table 3). Hemoglobin was measured using DxH 800 Hematology Analyser (Beckman Coulter). Total 25‐hydroxy vitamin D was measured using liquid chromatography tandem mass spectrometry. Other parameters were measured using Cobas analysers (Roche). Total protein intake expressed as g protein/kg per day was calculated using a 24‐hour urinary urea estimation (taking urea nitrogen to be 80% of total nitrogen), to support the validity of the estimated protein intake from the food diary, collected at the same time on day 1 of the assessment visit.

An audit of the diet related questions contained within the NAC's baseline preadmission questionnaire, provided additional depth to the nutritional overview (Table 5).

The AKUSSI was assessed as previously described employing standard items collected when patients attend for annual visits to the NAC.^22^

### 2.5 Statistical analysis

Variables are presented with their median, mean, SD, and interquartile range. Pearson's correlation coefficients and associated *P*‐values were calculated to assess the linear relationship between protein intake and severity of disease as defined by the AKUSSI score^6^ and the relationship of BMI with BMI calculated using the Powell‐Tuck and Hennessey equation (BMIPTH). All statistical analyses were performed with Stata version 15.1 (StataCorp. 2017. Stata Statistical Software: Release 15. College Station, TX: StataCorp LLC).

## 3 RESULTS

### 3.1 Body composition

**Figure 1:**
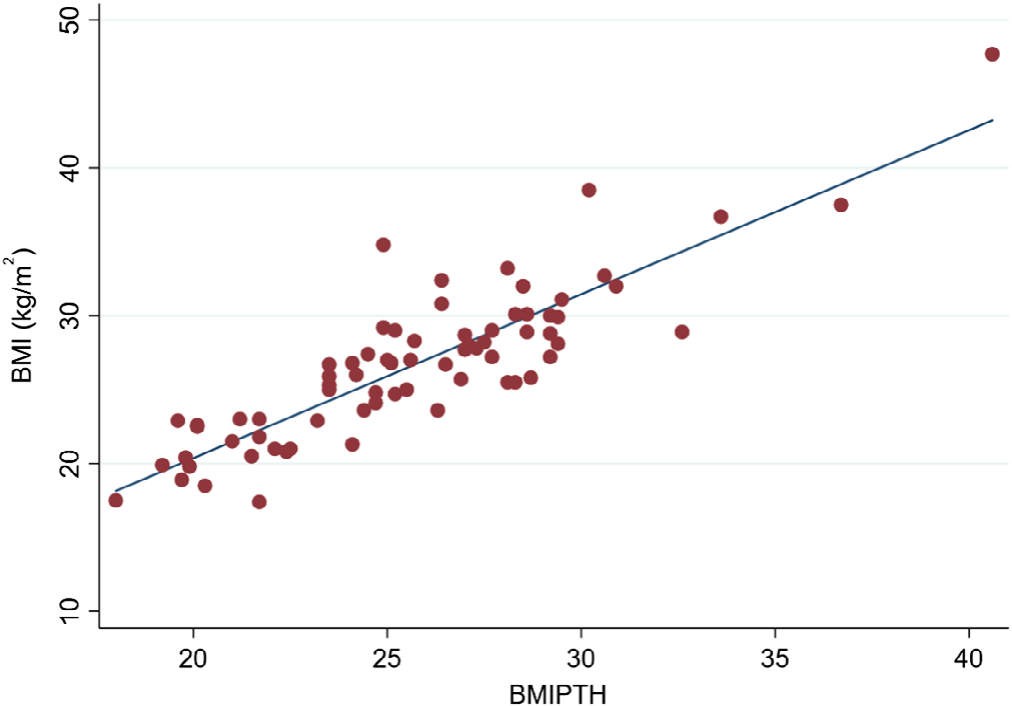
A scatter plot showing the relationship between observed BMI and BMIPTH values Pearson coefficient *r* (73) = .87, *P* < .001

See Table 1 and Figure 1.

### 3.2 Nutritional intake

**Figure 2:**
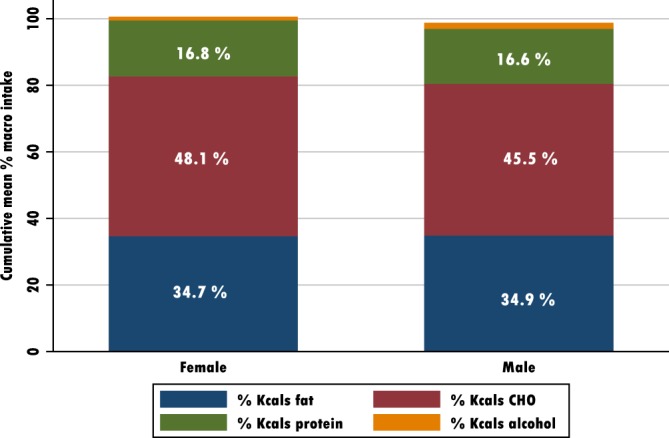
Comparing percentage energy contribution of macro nutrients between males and female AKU patients

See Tables 2 and 3, and Figure 2.

### 3.3 Blood and urine parameters

**Figure 3:**
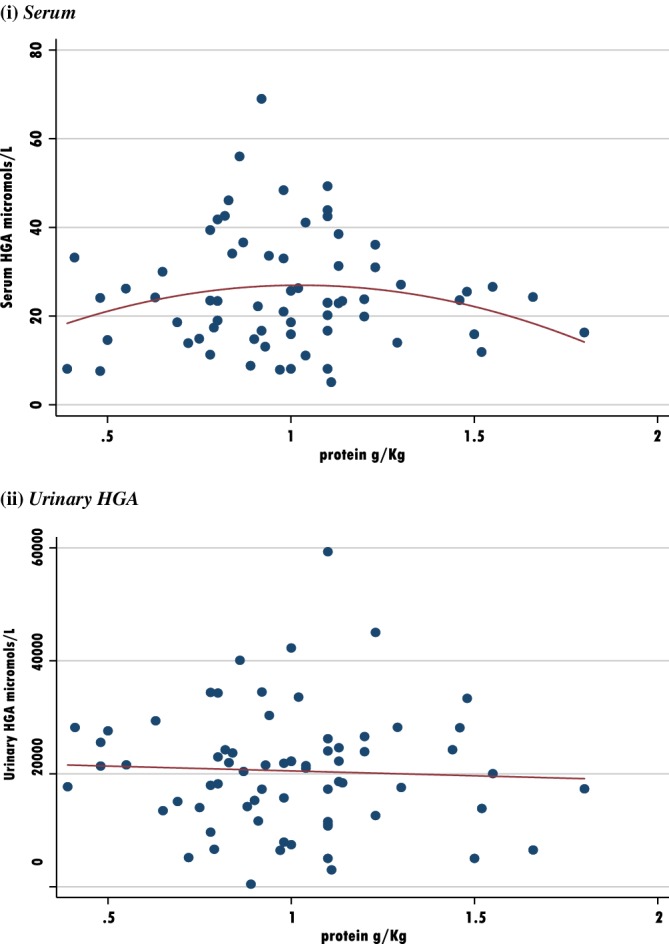
Scatter plots to assess impact of daily protein vs HGA. A, Serum; B, Urinary HGA

**Figure 4:**
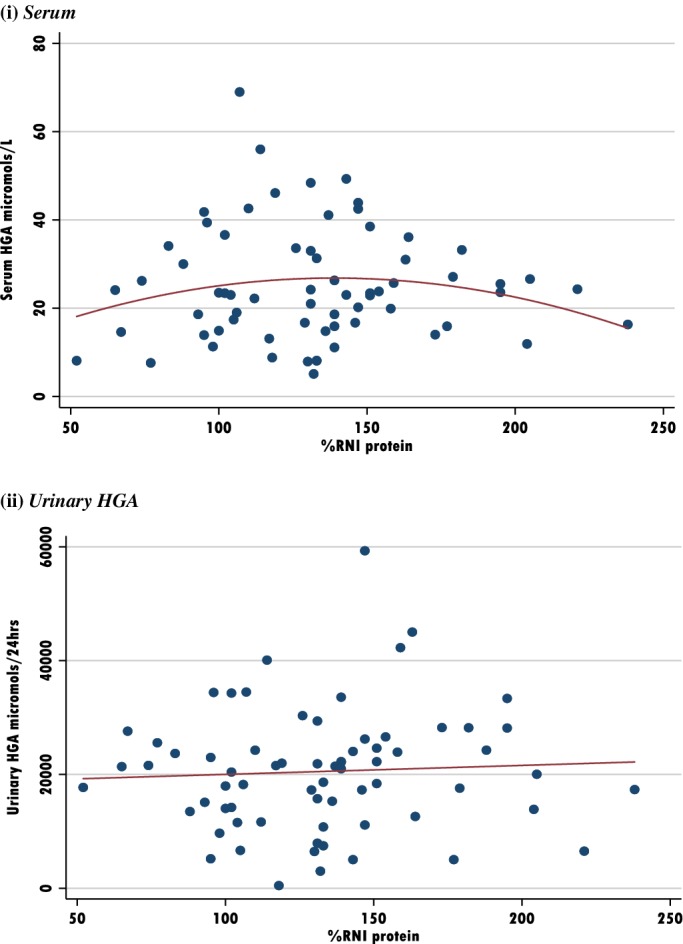
Scatter plots to assess the impact of Protein %RNI vs HGA. A, Serum; B, Urinary HGA

See Table 4 and Figures 3 and 4.

**Table 4:** Significant blood biochemistry—Vitamin D

25‐Hydroxy vitamin D (nmol/L)	Number	Median	IQR	Mean	SD	UK mean/% < 25 nmol/L
Male	25	35.0	25.0‐61.0	49.4	34.5	
Female	16	44.0	29.0‐85.0	56.8	38.8	
Males (age)						
19‐64	21	33.0	23.0‐51.0	55.0	35.1	42.4/22
≥65	3	89.0	50.0‐92.0	77.0	23.4	43.5/21
Females (age)						
19‐64	11	36.0	14.0‐63.0	42.4	30.5	45.3/15
≥65	4	103.5	77.0‐126.0	101.5	29.2	47.9/9

### 3.4 AKUSSI score—potential impact of diet

**Figure 5:**
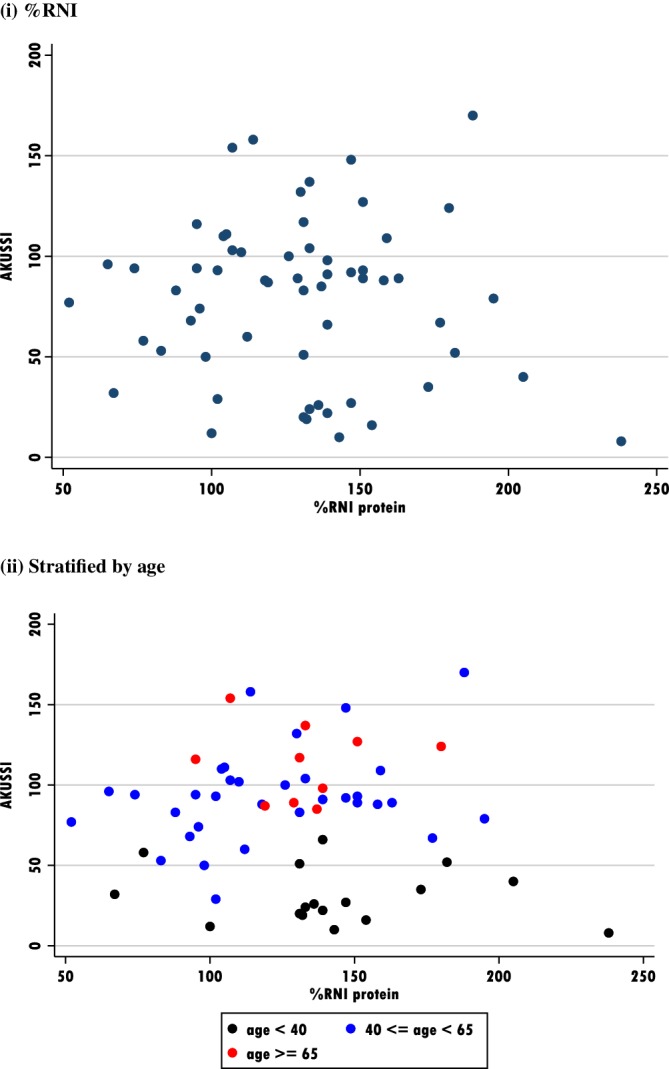
Scatter plots to consider protein intake vs AKUSSI score. A, %RNI; B, Stratified by age

**Figure 6:**
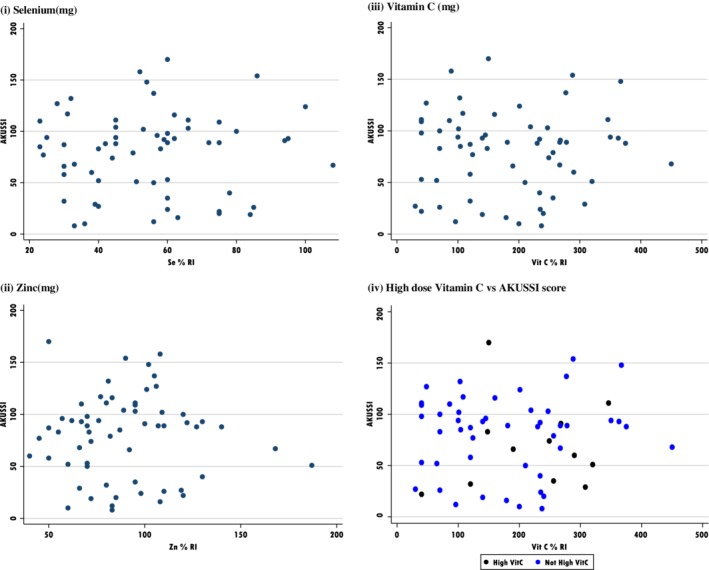
Scatter plots to consider intake of anti‐inflammatories vs AKUSSI score. A, Selenium (mg); B, Zinc (mg); C, Vitamin C (mg); D, High dose vitamin C vs AKUSSI score

See Figures 5 and 6.

### 3.5 Audit of diet related questions within AKU baseline questionnaire

See Table 5.

**Table 5:** Audit of diet related questions within AKU baseline questionnaire

N = 74	Number		Number	
Nos. reporting prior dietary intervention	35	High dose vitamin C	25	6 taking at visit 1
Received dietary advice unrelated to AKU	16 (11 for weight loss)	Glucosamine users	4	2 taking at visit 1
Self‐selected food manipulation	15 (14 vegetarian and 1 vegan)	Daily portions of fruit and vegetable	Male average 3	Female average 3.6
On a prescribed “diet” for AKU	17 (15 for low protein/2 for weight loss)	Over the counter nutritional supplement users	29	3 fish oils
Prescribed diet advised by a dietitian	7	Prescribed nutritional supplement	11	7 vitamin D_3_ (3 with calcium), 2 vitamin B_12_, and 2 vitamin C

### 3.6 Interpretation

#### 3.6.1 Body composition

All patients both male and female have a lower MUAC than the UK population with an IQR between the 5th and 25th centile. The mean for males across all ages at 30.2 cm is nearest to the 15th centile and is consistent across all ages. The mean female mid‐point MUAC of 29.6 cm is between the 25th and 50th centile. Bioimpedance data has not been used for comparative purposes using standard equations to provide a fat free mass index (FFMI) due to the potential inaccuracies of the height measure and lack of validity for non‐White ethnic groups or those with chronic disease (Franssen et al., 2014).^15^, ^16^, ^23^ The Tanita bioimpedance scale can underreport fat free mass particularly in low BMI.^24^, ^25^

#### 3.6.2 Grip strength

Male and female grip strength is not normally distributed. Male and female means (33.1 and 22.0 kg, respectively) are below 85% functionality, which is considered a poor outcome predictor postmajor surgery,^26^ an indicator of increased postoperative complications, increased length of hospitalization, higher rehospitalization rate, and decreased physical status and therefore a marker of poor nutritional status.^27^ Only one of the 34 men achieved the expectation of 40 kg. None of the 15 females achieved the expectation of 27.5 kg, but numbers are too small to stratify by age. No single individual achieved above average muscle functionality.

#### 3.6.3 Percentage body fat

Compared to the percentage of body fat within a nationally representative sample adjusted for ethnicity and age,^14^ the male AKU patients have a higher percentage fat. Centiles derived from bioimpedance measures,^15^, ^16^ indicates the male mean is on the 75th centile, with an IQR from the 50th to over the 95th centile. Numbers are too small to stratify by age, but suggests a consistent pattern of above average fat scores until the sixth decade when it falls to average norms then falls below expectations, suggesting a call on nutritional reserves at this point. Energy stores are depleted for the two patients in their 4th to 5th decade. Females consistently record lower energy intakes than UK mean, yet show a similar trend of lower than anticipated BMI and % body fat between the 4th and 5th decades.

#### 3.6.4 BMI

Sixty‐three percent of the adult UK populations are overweight or obese^28^ yet mean BMI for AKU men was 26.9 and women 26.3. National data would predict at least one man and two women within the morbidly obese range, whereas this cohort has only one female. Incidence of underweight is not normally distributed with a higher prevalence than the 2.3% national expectation, with trends indicating a fall in body weight during the 4th and 5th decade. BMI calculated using the Powell‐Tuck and Hennessey equation (Figure 1, BMIPTH) is highly correlated for this group, with a Pearson correlation coefficient of .86, indicating that although height loss is universal for this group BMI estimates are reliable, which is reassuring since measuring height is often painful due to ochronosis of the spine.

### 3.7 Nutritional intake

#### 3.7.1 Macronutrient intakes

Mean protein intake for men aged 19 to 64 years is 75.7 g compared to 67.4 g for women. Men aged ≥65 consume 71.0 g protein daily and 58.7 g for women, which are less as a percentage of total energy than the UK average (Table 2). A majority of the UK population takes more than 100% RNI, but in AKU 12% take less than the minimum 0.74 g protein/kg,^29^ with older women most at risk. The shortfall in protein intake is not fully compensated for by energy from fat, carbohydrate or alcohol, resulting in a theoretical energy deficit: 118 kcal/day across all patients and all ages, 404 kcal/day in men over 65 years with a relatively small SD and a mean of 112 kcal in women. Amino acid profiles of 50 of these AKU patients recorded mean phenylalanine, aspartic acid and arginine levels outside the normal reference range at initial assessment.^30^

There is a highly statistically significant association between the estimated protein intake from the food diary record (Table 3) and the 24 hours urinary nitrogen estimation (*r* (53) = .52, *P* < .001) suggesting the food diaries fairly reflect protein intake, which is irrespective of gender (*r* (34) = .67, *P* = .002 and *r* (18) = .48, *P* = .003 males/females, respectively).

The mean intake of protein expressed in g/kg is similar to the 1.0 g protein/kg reported in other AKU patients.^3^ Although not statistically significant, the estimated protein intake using urinary nitrogen trend was consistently lower for males and females across all age groups.

Total energy intake is consistently lower across all patients than reported national data, particularly among men. Older women take closer to the recommended 50% of total energy from carbohydrate than others, but with total fiber below average. This is most likely derived from nonextrinsic sugars, not untypical in older age choosing easy reach snack foods such as biscuits and cakes (Figure 2).

Overall alcohol intake is low in this group, with 68% abstaining from alcohol altogether compared to 15% of men and 21% of women nationally.^31^ The average percentage energy from alcohol is 3% for men and 0.4% for women, compared to 4.4% across all adults in the United Kingdom.

#### 3.7.2 Micronutrient

Intakes mirror those for the UK diet as a whole, with fewer portions of fruit and vegetables and total fiber. Those recording an LRNI are within normal population expectations. Wide standard deviations and small numbers prevent accurate interpretation by age. As a subgroup, there are 13 vegetarians and 1 vegan (all of Asian origin). The 11 men average 10.3 mg iron/day, just within the 10 to 15 mg daily range: the 3 women (averaging 10.7 mg) are well within national expectation (8‐13 mg/day).

### 3.8 Blood and urine measurements

#### 3.8.1 Serum albumin (g/L) and hemoglobin

Serum albumin (g/L) and hemoglobin concentration records are within national expectations and normally distributed for age and sex (Table 4). Prealbumin, as an inflammatory marker, is a valid predictor of nutritional risk^32^ and protein catabolism,^33^ not currently measured.

#### 3.8.2 Serum total 25‐hydroxy vitamin D (normal values above 50 nmol/L D2 + D3)

A mean of 49.4 nM for males, with no recorded level below 25 nM, compares to national levels. Females' record a mean of 56.8 nM compared with the national mean of 45 nmol/L, while none recorded less than 25 nM; the national expectation would be 15%—reflecting the frequency of prescribed supplementation of the 20% of AKU patients in this cohort of Asian origin.^34^

#### 3.8.3 Homogentisic acid

Figure 3 considers the potential impact of dietary protein intake on serum and urinary HGA as a %RNI and as g/kg actual weight. There appears to be a possible inverse quadratic relationship between protein intake and serum HGA. Patients with a protein %RNI > 150 or protein intake above 1 g/kg may experience lower serum HGA levels. All serum HGA < 40 for %RNI > 150. No differences were observed between serum HGA, when intakes of selenium, zinc or vitamin C were considered.

### 3.9 AKUSSI score

Mean AKUSSI and %RNI for protein by age (Table 6), shows an increasing score, but relatively stable protein intake. AKUSSI correlations have been undertaken to investigate the potential impact of protein expressed as g/kg and %RNI and stratified by age (Figure 5) and the potential impact of anti‐inflammatory nutrients (vitamin C, selenium, and zinc), alongside the potential impact of high dose vitamin C, given their historical potential significance in the literature (Figure 6). All demonstrate no association.

**Table 6:** Comparison of mean AKUSSI and %RNI for protein by age

Age	Mean AKUSSI (SD)	Mean %RNI protein (SD)
<40	30.5 (17.5)	146.3 (42.7)
40 ≤ 65	93.8 (28.7)	125.8 (36.6)
≥65	113.4 (23.2)	130.2 (21.6)

### 3.10 Audit overview

The preadmission questionnaire data (Table 5 **)** suggests many AKU patients have tried to manipulate their diet at some point in their lives. Limiting protein and supplements of vitamin C, glucosamine and/or chondroitin are the most frequent dietary approaches reported. Fifty percent of all patients reported some level of dietary protein manipulation over their lives, 42% of which recorded a low protein diet, yet less than half had been referred to a dietitian for dietary guidance. Twenty percent of patients report being “on a diet for AKU” at visit 1.

## 4 DISCUSSION

This is the most comprehensive study of the nutritional status of people with AKU reported in the literature. Evidence collected, using a variety of valid nutritional surveillance techniques, provides the opportunity to consider the overall nutritional status, which is hitherto unreported in this genetic condition. Those with AKU have frequently been advised to manipulate their diets at some point in their lives (Table 5), based on the limited anecdotal evidence from the literature, which includes limiting total protein intake and taking prophylactic doses of vitamin C. The lack of correlation between the AKUSSI score and intakes of protein, expressed as either %RNI or as g/kg or any anti‐inflammatory nutrients, when stratified by age, provides further evidence of the inappropriateness of dietary manipulation as treatment for this condition.^1^, ^35^

AKU patients have lower muscle mass and higher % body fat as evidenced through bioimpedance, significantly lower grip strength, MUAC and BMI across all age groups. This suggests a chronically protein‐depleted diet, best described as under‐nourished^36^ or more accurately “diet related malnutrition without inflammation” termed DRM (ESPEN, 2017^37^).

Previous attempts to control weight to minimize the impact of ochronosis on joints and cartilage could have had an impact in addition. Reduced muscle stores and muscle functionality, results in a reduced basal metabolic rate. This in turn makes weight gain more likely, seen here as a higher % body fat. With less metabolically active tissue and reduced mobility associated with the joint and cartilage destruction, there is a risk of obesity over time, increasing the burden of disease.

The nutritional analyses indicates a lower than average protein intake than the UK norm, but it cannot be considered “low” in a therapeutic sense and the food diary itself reflects only a moment in time as opposed to a life‐time dietary behavior. Although the average estimated protein intake of 1 g/kg body weight is within the recommended range, two of the seven patients with recorded protein intakes below the minimum 0.74 g/kg, were vegetarian, suggesting an additional over‐arching risk. This consistently near minimum protein intake provides little scope for episodes of increased need over the life course.^38^

The possibility of the condition itself having an impact on muscle development must also be considered, since phenylalanine and tyrosine are proteinogenic amino acids, with tyrosine a precursor of melanin, epinephrine and thyroid hormones.^39^

Patients with AKU frequently require joint replacements by their fourth and fifth decade. Some have undergone often multiple joint replacements prior to visiting the NAC. These periods of high protein/energy requirements, create pinch points of higher metabolic demand impacting on muscle and fat reserves and consequently BMI, potentially explaining why observed trends in obesity regress during the 5th and 6th decade, when joint replacements are routine treatment strategies. This in turn represents an additional risk for patients with AKU, for extended bed stay and adverse outcome associated with malnutrition.^40^ The lower total energy intake and reduced mobility associated with the condition itself risks muscle atrophy, as a result of increased breakdown and decreased synthesis.^41^

HGA‐lowering therapy, using nitisinone, is now a possibility in AKU treatment.^22^ Nitisinone has been used as the standard of care in hereditary tyrosinaemia 1 for more than 20 years with remarkable success, transforming the care of this previously fatal tyrosine pathway disorder. Nitisinone inhibits p‐hydroxyphenylpyruvate dioxygenase, thereby decreasing distal metabolites including HGA. While this HGA‐lowering effect is beneficial in AKU, by introducing a proximal metabolic block, nitisinone results in tyrosinaemia.^42^ Postnitisinone tyrosinaemia has been associated with unwanted corneal keratopathy (with a potential to cause blindness), cutaneous rashes, and cognition effects, which can be mitigated by a lower dietary tyrosine intake.^23^

### 4.1 Implications for future clinical practice

Manipulating protein and energy intake as a consequence of treating AKU presents a challenging therapeutic demand. Dietary strategies need to support adequate nutritional intake, limit protein, yet maintain already depleted muscle mass, even though exercise tolerance may be compromised, limit further fat accumulation and maintain nutritional status during potentially repeated episodes of increased metabolic demand, while maintaining a safe serum tyrosine.

### 4.2 Therapeutic considerations

1. Bioimpedance measures (BIA) augment standard anthropometry to monitor body composition trends, particularly muscle and fat mass, since joint pain in AKU affects use of dynamometers and scoliosis affects skin fold points.
2. Estimate BMI using a MUAC,^13^ as a simple, noninvasive measure, since ochronosis of the spine makes measuring height painful.
3. Strategies to maintain integrity of lean body mass, such as resistance exercise to help minimize the risk of muscle atrophy and sarcopenia associated with older age.^43^
4. Include serum prealbumin measurement as part of routine biomedical analysis.

### 4.3 Areas of future service development

1. Establish a robust pre‐ or peri‐operative feeding regime prior to joint replacement surgery to ensure AKU patients are not included in the 60% elderly patients who are protein‐energy undernourished on hospital admission or develop serious nutritional deficits delaying discharge.
2. A strategic cascade of this body composition data across the AKU community, particularly parents and careers of children with AKU, to raise awareness of the risk of inadequate protein/energy intake during the growing years.

### 4.4 Limitations of this study

1. Using bioimpedance to measure body composition is less accurate than gold standard DEXA scan for body composition analysis,^12^, ^13^ as it may overestimate fat free mass compared with DXA.^25^, ^44^
2. The digital grip strength dynamometer requires validation against the gold standard Jamar hand grip dynometer (Patterson Medical).
3. Using NHANES study reference data to interpret percentage body fat as their multiethnic population differs from this group.^45^, ^46^

#### AUTHOR CONTRIBUTORSHIP

S. Judd carried out all of the nutritional assessments and wrote the first draft. A. M. Milan, A. T. Hughes, and A. S. Davison undertook the biochemical analyses. E. E. Psarelli and A. Needham undertook the statistical analysis. L. R. Ranganath, A. Shenkin, M. Khedr, and A. S. Davison provided intellectual input and editorial support.

## CONFLICT OF INTEREST

S. Judd, M. Khedr, A. M. Milan, A. S. Davison, A. T. Hughes, E. E. Psarelli, A. Needham, L. R. Ranganath, and A. Shenkin declare that they have no conflicts of interest of any kind.

## DETAILS OF FUNDING

The National Alkaptonuria Service is funded by the Highly Specialised Services National Health Services England.

## COMPLIANCE WITH ETHICS GUIDELINES

All procedures reported in this review were in accordance with the ethical standards of the local Hospital ethics committee and with the Helsinki Declaration of 1975, as revised in 2000. The National Alkaptonuria Centre and all subsequent data analysis has been approved by the Royal Liverpool and Broadgreen University Hospital Trust Audit Committee (Audit no. ACO3836).

## A PATIENT CONSENT STATEMENT

Informed consent was obtained from all patients for being included in the study. This is being published as a clinical practice article and standard research ethics process is not therefore appropriate. The data obtained were following standard clinical assessments upon referral to the National Alkaptonuria Service in Liverpool. Patients are informed verbally and through being handwritten materials about the activities of the National AKU Service. They are explicitly informed in the Patient information booklet of the National AKU Service that: We could publish results from the study but if we do, we will make sure you cannot be identified in any way. All data used for publicity or for other research purposes will ensure total anonymity. Please let us know when you are visiting Ward 9 B (where the National AKU Service is located) that you understand this and have no objection to it.

## ANIMAL RIGHTS

This article does not contain any studies with human or animal subjects, performed by any of the authors.

## Supporting information


**Data S1** Supporting information file.

## Data Availability

The data that support the findings of this study are available from the corresponding author upon reasonable request.
