# Supplementary material for: The nutritional status of people with alkaptonuria: An exploratory analysis suggests a protein/energy dilemma
Source: JIMD Rep. 2020 Mar 17;53(1):45–60. doi: 10.1002/jmd2.12084 (PMC7203650; doi:10.1002/jmd2.12084)
Supplement: Supplementary file 1 — Data S1 Supporting information file. [file JMD2-53-45-s001.doc]

**DEPARTMENT OF NUTRITION AND DIETETICS**

**ROYAL LIVERPOOL AND BROADGREEN UNIVERSITY HOSPITAL**

**Prescot Street**

**Liverpool**

**L7 8XP**

**FOOD DIARY**

**Your name: ………………………………………………………………**

**Please complete this food diary for 7 days**

**Please follow the following instructions when filling in this diary to help you achieve as accurate a result as possible.**

1. Use this diary to write down **everything** that you have to eat and drink each day.
2. Try to fill in this diary at the time of having food or drinks, instead of leaving it for the end of the day.
3. Start each day on a new page and fill in your name and date on top of the page.
4. Provide as much detail as possible about the types, amounts and preparation methods of food or drinks that you have:

**Include the following information where possible:**

- brand names, e.g. yoghurt: Muller, Weight Watchers, etc
- home made or ready made
- cooking method, e.g. roasted, boiled, stewed, or grilled
- preparation method, e.g. meat: fat cut off, potatoes: mashed
- whether the food was fresh, frozen or tinned
- type of food, e.g. low fat, light, sugar-free, low salt
- amount or portion size, e.g. 2 small scoops of mash potato
- weight, if available, e.g. from packaging
- any additions to the food, e.g salt, mayonnaise, salad dressing

1. Provide details of ‘made up’ dishes, e.g.:
   - cheese sandwich: 2 slices of thick cut wholemeal bread, butter thinly spread, thin slices of cheese
   - Stew: lean beef, onions, carrots, potatoes, canned tomatoes

**Please answer the following questions and tick the correct box:**

1. How much milk do you use:

in tea……………………....

in coffee……………………

other………………………..

none 

1. How much sugar do you use:

in tea………………………..

in coffee…………………….

other………………………..

none 

1. What type of milk do you use:

 full cream

 semi-skimmed

 skimmed

 other, specify……………………………………………………………

1. What type of bread do you use:

 white

 wholemeal

 best of both

 other, specify……………………………………………………………..

1. What brand of bread do you use?................................................................................
2. What thickness of bread do you choose:

 thick cut

 medium cut

 thin cut

 barms / rolls

1. If you use fat spread, which one do you use?

 butter

 margarine

 low fat spread

 other, please specify………………………………………………………….

1. Which brand of fat spread do you use?.............................................................................
2. If you fry any food, which type of fat do you use?.........................................................
3. Are you taking a vitamin /mineral/fish oil supplement? Yes/No. If yes which ones and how often are you taking them?.........................................................................
4. What is your current weight?.........................................................

**SAMPLE MENU**

Name……………………………………………….Date…………………………..

| **TIME** | **FOOD/DRINK TAKEN** | **AMOUNT TAKEN** | **DESCRIPTION OF FOOD /**  **OTHER INFORMATION** |
| --- | --- | --- | --- |
| **Morning**  **8:30** | Orange juice  Rice Crispies  Sugar  Wholemeal bread  Butter  Tea with milk, no sugar | 1 small glass  4 tablespoons  1 teaspoon  1 thin slice  thickly spread  1 cup | No added sugar |
| **During the morning**  **11:00** | Low fat yoghurt | 1 small pot | Muller Light  (100g) |
| **Mid-day**  **12:30** | Wholemeal bread  Margarine  Ham  Apple  Diet lemonade | 2 thin slices  thinly spread  2 slices  1 medium  1 can |  |
| **During the afternoon**  **16:00** | Tea with milk, no sugar  Digestive biscuits | 1 mug  2 plain |  |
| **Evening**  **19:00** | Pork chop  Potatoes  Carrots  Peas  Pineapple  Custard | 2 small  4 small  4 tablespoons  3 tablespoons  3 rings  1 mug | Fried in vegetable oil, fat removed  Boiled, no salt  Boiled  Boiled  Tinned in syrup  Made with full cream milk |
| **During the evening**  **21:30** | Milk  Crumpet  Margarine  Jam | 1 cup  2 toasted  thinly spread  2 teaspoons | Full cream milk  Reduced sugar jam |

**FOOD DIARY**

Name………………………………………………….Date…………………………..

| **TIME** | **FOOD/DRINK TAKEN** | **AMOUNT TAKEN** | **DESCRIPTION OF FOOD /**  **OTHER INFORMATION** |
| --- | --- | --- | --- |
| **Morning** |  |  |  |
| **During the morning** |  |  |  |
| **Mid-day** |  |  |  |
| **During the afternoon** |  |  |  |
| **Evening** |  |  |  |
| **During the evening** |  |  |  |

**FOOD DIARY**

Name………………………………………………….Date…………………………..

| **TIME** | **FOOD/DRINK TAKEN** | **AMOUNT TAKEN** | **DESCRIPTION OF FOOD /**  **OTHER INFORMATION** |
| --- | --- | --- | --- |
| **Morning** |  |  |  |
| **During the morning** |  |  |  |
| **Mid-day** |  |  |  |
| **During the afternoon** |  |  |  |
| **Evening** |  |  |  |
| **During the evening** |  |  |  |

**FOOD DIARY**

Name………………………………………………….Date…………………………..

| **TIME** | **FOOD/DRINK TAKEN** | **AMOUNT TAKEN** | **DESCRIPTION OF FOOD /**  **OTHER INFORMATION** |
| --- | --- | --- | --- |
| **Morning** |  |  |  |
| **During the morning** |  |  |  |
| **Mid-day** |  |  |  |
| **During the afternoon** |  |  |  |
| **Evening** |  |  |  |
| **During the evening** |  |  |  |

**FOOD DIARY**

Name………………………………………………….Date…………………………..

| **TIME** | **FOOD/DRINK TAKEN** | **AMOUNT TAKEN** | **DESCRIPTION OF FOOD /**  **OTHER INFORMATION** |
| --- | --- | --- | --- |
| **Morning** |  |  |  |
| **During the morning** |  |  |  |
| **Mid-day** |  |  |  |
| **During the afternoon** |  |  |  |
| **Evening** |  |  |  |
| **During the evening** |  |  |  |

**FOOD DIARY**

Name………………………………………………….Date…………………………..

| **TIME** | **FOOD/DRINK TAKEN** | **AMOUNT TAKEN** | **DESCRIPTION OF FOOD /**  **OTHER INFORMATION** |
| --- | --- | --- | --- |
| **Morning** |  |  |  |
| **During the morning** |  |  |  |
| **Mid-day** |  |  |  |
| **During the afternoon** |  |  |  |
| **Evening** |  |  |  |
| **During the evening** |  |  |  |

**FOOD DIARY**

Name………………………………………………….Date…………………………..

| **TIME** | **FOOD/DRINK TAKEN** | **AMOUNT TAKEN** | **DESCRIPTION OF FOOD /**  **OTHER INFORMATION** |
| --- | --- | --- | --- |
| **Morning** |  |  |  |
| **During the morning** |  |  |  |
| **Mid-day** |  |  |  |
| **During the afternoon** |  |  |  |
| **Evening** |  |  |  |
| **During the evening** |  |  |  |

**FOOD DIARY**

Name………………………………………………….Date…………………………..

| **TIME** | **FOOD/DRINK TAKEN** | **AMOUNT TAKEN** | **DESCRIPTION OF FOOD /**  **OTHER INFORMATION** |
| --- | --- | --- | --- |
| **Morning** |  |  |  |
| **During the morning** |  |  |  |
| **Mid-day** |  |  |  |
| **During the afternoon** |  |  |  |
| **Evening** |  |  |  |
| **During the evening** |  |  |  |
